# Convergent Differential Regulation of Parvalbumin in the Brains of Vocal Learners

**DOI:** 10.1371/journal.pone.0029457

**Published:** 2012-01-06

**Authors:** Erina Hara, Miriam V. Rivas, James M. Ward, Kazuo Okanoya, Erich D. Jarvis

**Affiliations:** 1 Department of Neurobiology, Howard Hughes Medical Institute, Duke University Medical Center, Durham, North Carolina, United States of America; 2 Graduate School of Advanced Integration Science, Chiba University, Chiba, Japan; 3 Laboratory for Biolinguistics, RIKEN BSI, Saitama, Japan; University of Lethbridge, Canada

## Abstract

Spoken language and learned song are complex communication behaviors found in only a few species, including humans and three groups of distantly related birds – songbirds, parrots, and hummingbirds. Despite their large phylogenetic distances, these vocal learners show convergent behaviors and associated brain pathways for vocal communication. However, it is not clear whether this behavioral and anatomical convergence is associated with molecular convergence. Here we used oligo microarrays to screen for genes differentially regulated in brain nuclei necessary for producing learned vocalizations relative to adjacent brain areas that control other behaviors in avian vocal learners versus vocal non-learners. A top candidate gene in our screen was a calcium-binding protein, parvalbumin (PV). In situ hybridization verification revealed that PV was expressed significantly higher throughout the song motor pathway, including brainstem vocal motor neurons relative to the surrounding brain regions of all distantly related avian vocal learners. This differential expression was specific to PV and vocal learners, as it was not found in avian vocal non-learners nor for control genes in learners and non-learners. Similar to the vocal learning birds, higher PV up-regulation was found in the brainstem tongue motor neurons used for speech production in humans relative to a non-human primate, macaques. These results suggest repeated convergent evolution of differential PV up-regulation in the brains of vocal learners separated by more than 65–300 million years from a common ancestor and that the specialized behaviors of learned song and speech may require extra calcium buffering and signaling.

## Introduction

**Figure 1:**
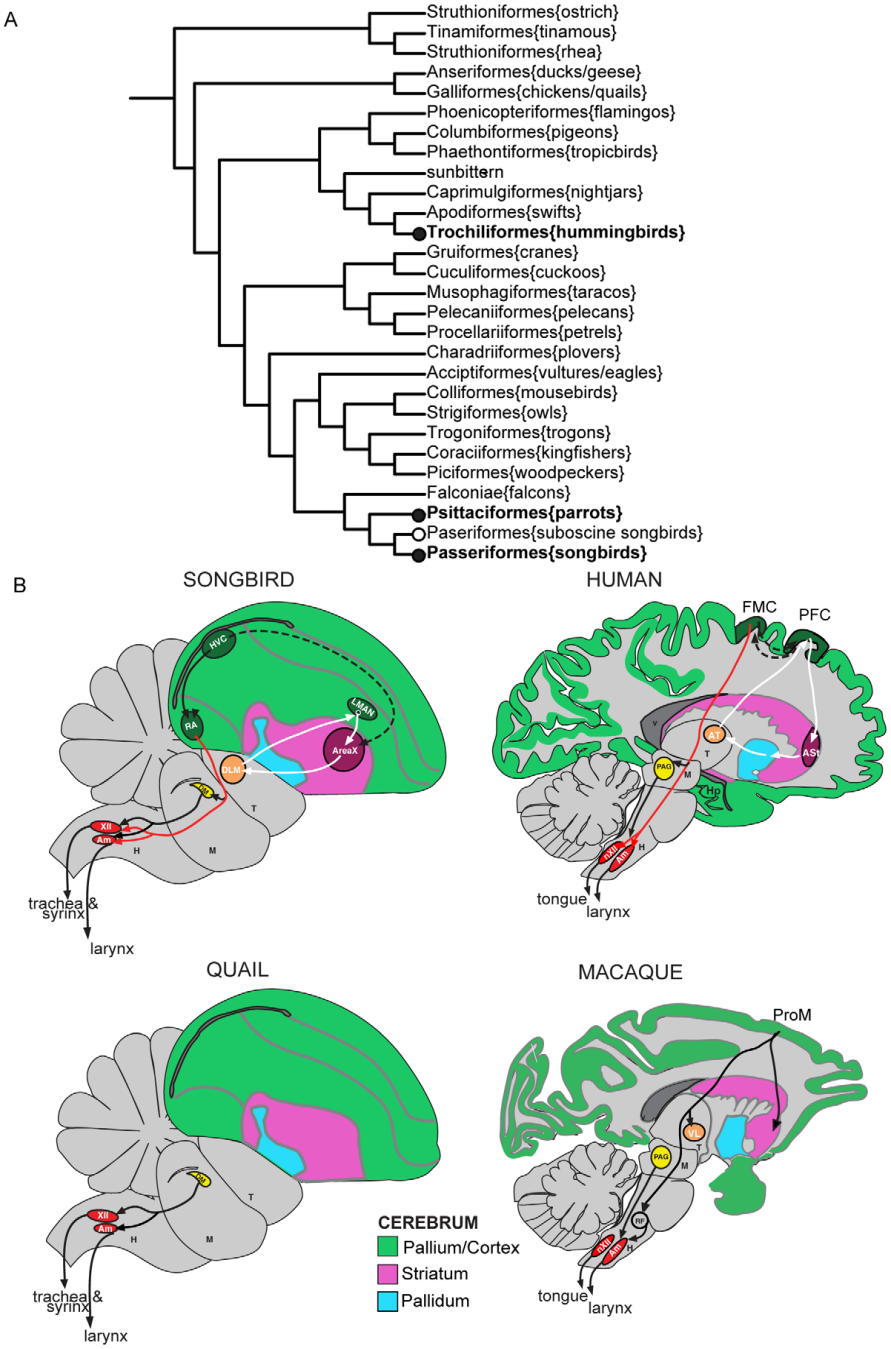
Avian phylogenic tree and schematic drawings of vocal learner and non-learner brains. (A) Avian phylogenic tree. Shown are the branches for 27 major orders and one suborder (suboscines), highlighting 1–2 species each, based on the proposal of Hackett et al 2008 [64]. Bold text, vocal learners. Black nodes, proposed independent gains of vocal learning. White node, an alternative possibility where there was two independent gains of vocal learning (hummingbirds and the common ancestor of parrots and songbirds), then lost in suboscine songbirds. (B) Schematic sagittal drawing of example vocal learner (songbird and human) and non-learner (quail and macaque) brains. Black lines, song motor pathway. White lines, pallial-basal-ganglia song pathway. Dashed lines, connections between the two pathways. Red line, direct projection from forebrain to brainstem vocal motor neurons found in vocal learners. Connections in humans are predicted based on known motor pathways in mammals, except the direct projection to Amb and nXII, which has been experimentally determined in humans. Non-human primates have what is called a pro-motor (ProM) region (or laryngeal motor cortex) in the premotor cortex that makes an indirect projection to Amb, but unlike vocal learners this region is not required nor appears to influence vocalizations. For reviews, see Jurgens (2002) [6], Jarvis (2004) [2], Fitch et al (2010) [7], and Simonyan et al (2011) [65]. Abbreviations: Am or Amb, nucleus ambiguus Area X, a vocal nucleus (no abbreviation) ASt, anterior striatum AT, anterior thalamus DLM, dorsal lateral nucleus of the thalamus DM, dorsal medial nucleus of the midbrain FMC, face motor cortex H, hindbrain HVC, a vocal nucleus (no abbreviation) LMAN, lateral magnocellular nucleus of the anterior nidopallium M, midbrain, nXII, 12^th^ motor nucleus PAG, periaqueductal gray PFC, prefrontal cortex ProM, promoter laryngeal cortex in non-human primates RA, robust nucleus of the arcopallium RF, reticular formation T, thalamus V, ventricle.

Vocal learning is a rare trait found in only a few species of mammals (humans, cetaceans, bats, elephants, and sea lions) and three groups of birds (songbirds, parrots, and hummingbirds) [1], [2]. It is a critical behavioral substrate for spoken-language in humans and song in song learning birds [3], [4]. Because this trait and associated brain pathways are not found in species more closely related to each vocal learning order, it has been argued that vocal learning evolved independently in each lineage (Fig. 1A) [2], [5]. Yet vocal learning species share similar communication features, such as a requirement for auditory feedback to develop and maintain learned vocalizations, vocal learning critical periods, cultural transmissions of vocal repertoires, and specialized forebrain pathways that make a unique projection to brainstem vocal motor neurons, all of which have so far not been found in vocal non-learners (Fig. 1B) [2], [3], [6], [7].

Vocal learning brain pathways have been best characterized in avian vocal learners, and consist of two sub-pathways: a posterior song motor pathway involved in production of learned song and an anterior pallial-basal-ganglia-thalamic loop involved in song learning [2], [8], [9], [10], [11]. The posterior pathway contains the arcopallium song nucleus (songbird robust nucleus of the arcopallium [RA], parrot central nucleus of the anterior arcopallium [AAc], hummingbird vocal nucleus of the arcopallium [VA]), which makes a direct projection to the brainstem vocal motor nucleus, the tracheasyringeal portion of the 12^th^ motor nucleus, abbreviated nXllts. This connection is similar to humans, where the face motor cortex makes a direct projection to the mammalian analog of brainstem vocal motor neurons, nucleus ambiguus (Amb) [2], [6], [7], [8], [11], [12]. The avian nXllts innervates the syrinx and avian and mammalian Amb innervates the larynx (Fig. 1B) [11]. However, it is not known if the avian larynx contributes to vocalizations as it does in mammals; instead in birds, the syrinx is the major organ that generates vocalizations. The caudal portion of nXII in birds is nXIIts and the rostral portion of nXII innervates the tongue; all of nXII in mammals innervates the tongue, which in humans also receives a direct projection from the face motor cortex [6]. This direct projection to nXIIts song learning birds and to Amb in humans is thought to be a defining feature that led to voluntary fine motor control over vocalizations and thus the evolution of song and spoken-language, respectively [2], [7], [12], [13]. Vocal non-learning birds and non-human mammals also possess the brainstem vocal motor and tongue motor neurons, but such neurons do not receive a direct projection from the forebrain in these species, except for a weak projection to nXII motor neurons in old world primates, including macaques [6], [14], [15], [16], [17], [18]. The vocal motor neurons in vocal non-learners are thought to be mainly connected in a conserved brainstem network that produces innate calls (Fig. 1B). Although vocal non-learning relatives do not have a forebrain vocal learning pathway, such pathways in song learning birds and humans have been proposed to have emerged from a pre-existing adjacent motor control pathway found in vocal learners and non-learners, indicating a possible deep homology of a motor learning brain pathway for their convergent evolution [19]. For example, the intermediate arcopallium (iA) laterally adjacent to RA is activated by non-vocal movement behaviors, and this part of iA makes descending projections to the brainstem and spinal cord reticular formation neurons, which in turn project to the motor neurons that control muscles for body movements [11], [19], [20], [21].

We hypothesize that the convergent behavioral and neuroanatomical features among vocal learning birds and humans are associated with specialized genetic changes in genes that develop and maintain vocal learning circuits. Partly consistent with this hypothesis, prior studies have identified specialized expression of several genes in forebrain song nuclei of avian vocal learners, such as glutamate and dopamine neurotransmitter receptors, cell adhesion molecules, calcium binding proteins, and transcription factors [22], [23], [24], [25], [26], [27], [28], [29], [32]. However, except for weak convergent higher NR2A glutamate receptor and FoxP1 transcription factor expression in the HVC analog of all three vocal learning bird lineages [23], [24], the differential expression patterns of other genes were either not tested across vocal learning lineages or if tested, were not found across all vocal learning lineages. Differential expression of genes has also been searched for in speech areas of human brains [30], [31], but these genes had not been assessed in birds. In humans, possible differential expression for two genes implicated in language acquisition and production, FOXP1 and FOXP2, have not yet been well assessed in brain speech areas [25], [33], [34].

In the present study, we used oligo nucleotide microarrays to address our hypothesis by performing a systematic screen for potential molecular differences in the two neuron populations that have the most unique connectivity difference between vocal learners and non-learners: the arcopallium song nucleus of vocal learners and its target, the nXIIts. After analyses in birds, we compared expression by in-situ hybridization in the nXII and Amb of humans and a non-human primate, macaques. Here we report on one of our top candidate genes, the calcium binding protein, parvalbumin (PV), which was also previously shown to be higher in songbird RA relative to the surrounding arcopallium [22], [26]. We found that PV was significantly up-regulated throughout the vocal motor forebrain pathway in species of all three avian vocal learning lineages, as well as in the brainstem vocal motor neurons of vocal learning birds and humans, but not in avian vocal non-learners and macaques. Based on known functions of PV [35], [36], [37], our findings suggest repeated selection in the evolution of vocal learning.

## Results

Using a laser capture microdissection microscope, we dissected: (1) the arcopallium song nucleus of vocal learners (songbird; zebra finches [*Taeniopygia guttata*] RA, parrot: budgerigars [*Melopsittacus undulatus*] AAc, and hummingbird: [*Calypte anna*] VA); (2) the equivalent location, the central intermediate arcpallium (ciA) in the middle of iA, in vocal non-learners (ring dove [*Stereptopelia capicola*] and Japanese quail [*Coturnix japonica*]); (3) the iA ventral-lateral to RA (or medial in parrot and hummingbird) in vocal learners; (4) the equivalent iA ventral-lateral to ciA in vocal non-learners; 3) the brainstem vocal motor neurons nXIIts in vocal learners and non-learners; and 4) the supraspinal (SSp) neck motor neurons in both groups. The arcopallium song nucleus among vocal learners is considered analogous based on its presence in the arcopallium, connectivity, developmental profile, gene expression profiles, and function [10], [23], [38], [39], [40], [41], [42]. The adjacent iA, unlike the arcopallium song nuclei, in both vocal learners and non-learners projects indirectly to motor neurons, via the reticular formation neurons [16], [20], [21], [43]. The nXIIts and SSp are present in all birds examined to date. However, like many other motor neurons, SSp does not receive direct projections from the forebrain (from the arcopallium) in vocal learners and non-learners thus far tested [20], [21].

From the laser captured cells, we isolated RNA, synthesized Cy3-labeled cDNA, and hybridized them in one-color Cy3 reactions to a custom-designed songbird oligonucleotide microarray that detects up to 44,000 transcripts ([44]; see methods). Our rational for choosing laser capture microscopy was to have anatomical regional specificity and accuracy. The values were normalized using median centered log2 transformation (see methods). Raw microarray data were deposited in GEO database (accession # GSE28395 and GES33667). The rationale for using microarrays to measure mRNA as opposed to other approaches, such as high-throughput proteomics, was that it is much more efficient and feasible to measure 10,000 s of mRNAs simultaneously than the limited number of proteins by today's technology (but see [45]). We have found that in the past, about 85% of the mRNA differences from our microarrays verified by in-situ hybridization were recapitulated at the protein level [46].

**Figure 2:**
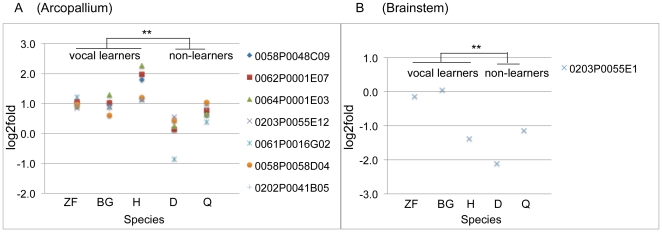
Microarray results for PV expression. (A) Seven different PV oligos (symbols) in the top 100 candidate gene transcripts were differentially expressed between vocal learners and non-learners in the arcopallium. (B) Example PV oligo that showed lower PV expression in nXllts compared to SSp in hummingbird, dove, and quail. Y-axes, log2 fold expression ratio of the arcopallium song nucleus or ciA versus the adjacent iA, or SSp relative to nXIIts; ratio = 0 means no difference between brain areas. Clone IDs of cDNAs that the oligos recognize are in http://songbirdtranscriptome.net/and http://aviangenomes.org. ZF, zebra finch; BG, budgerigar; H, hummingbird; D, dove; Q, quail. **p<0.01 paired t-test on each oligo of each group of species that share a trait (n = 9 vocal learners; n = 5 vocal non-learners; empirical Bayes adjusted paired t-test; the data shown is for averages of each species). **p<0.01, unpaired *t*-test, combined values of PV oligo variants.

An empirical Bayes paired t-test analysis between the arcopallium song nucleus and adjacent iA of each vocal learning species versus ciA and adjacent iA of vocal non-learning species (see methods) yielded lists of transcripts whose expression significantly differed in the arcopallium song nuclei of vocal learners relative to ciA of non-learners. In the list, seven out of 12 different oligos that measure putative PV splice variants were within the top 100 candidate gene transcripts that showed up-regulation in the arcopallium song nucleus of vocal learners, whereas there were either no or smaller differences in PV expression in the ciA versus adjacent iA of vocal non-learners. When we combined values of the seven different variants in unpaired *t*-test test, a significant difference between vocal learners and non-learners remained (Fig. 2A; *above values). PV was also within the top 100 candidate genes in nXIIts, but in the reverse direction with down regulation in nXIIts of vocal non-learners and the hummingbird relative to SSp (Fig. 2B). Detailed population analyses of other regulated genes will be presented in a separate study. Differential PV expression had been noted in songbird RA previously [22], but a systematic analysis across independent lineages of vocal learners and non-learners had not been conducted. We thus explored PV expression further.

### PV mRNA expression is specialized in forebrain song nuclei of avian vocal learners

**Figure 3:**
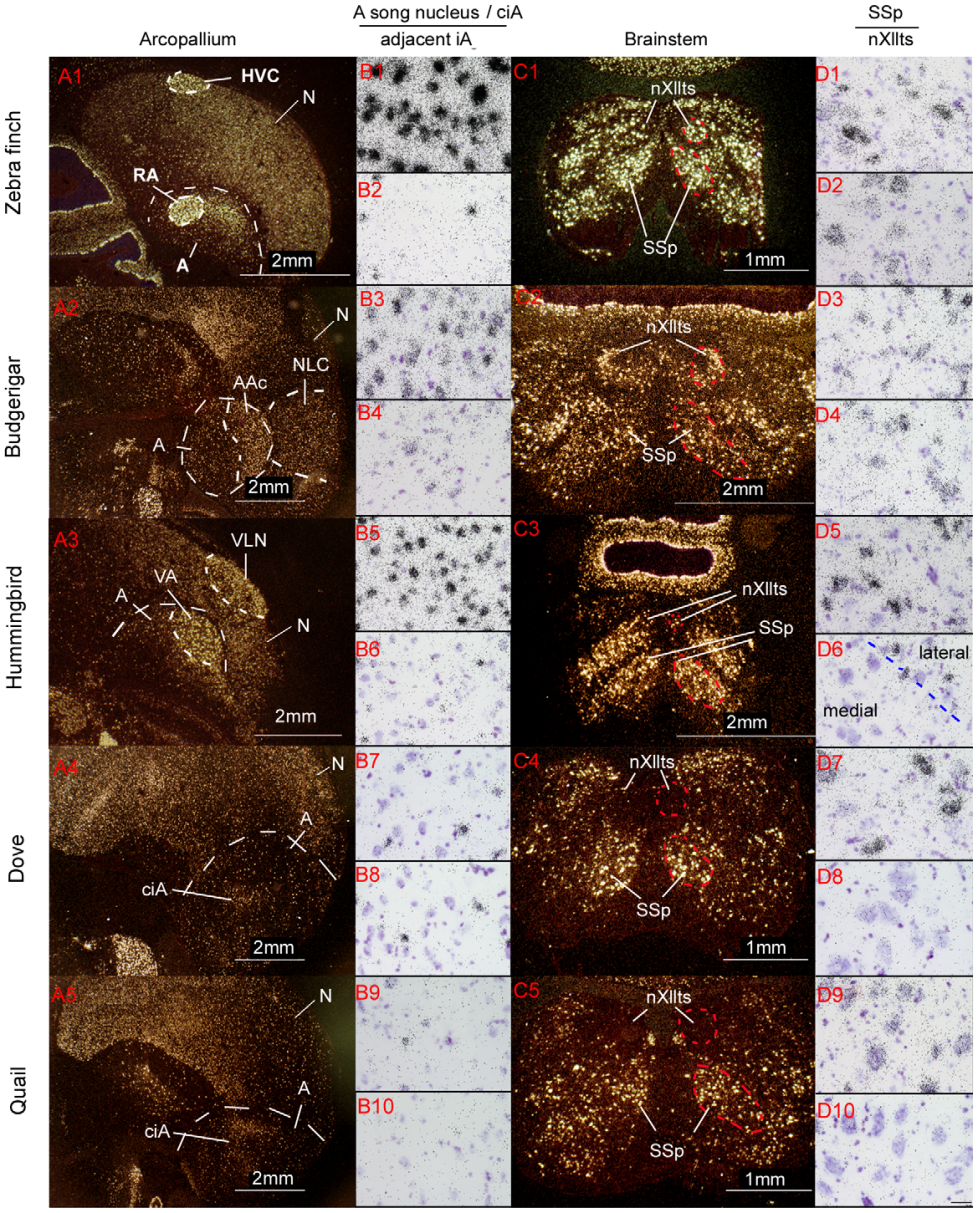
PV mRNA expression in vocal motor pathway nuclei in avian species. (A1–5) Darkfield images of PV expression in the arcopallium (delineated by dashed white lines) of three vocal learners and two non-learners. The arcopallium song nuclei are highlighted (RA, AAc, and VA) as well as the iA region lateral or medial used to compare with in the microarray and in-situ hybridization experiments. The nidopallium song motor nucleus (HVC, NLC, and VLN) of each species is also highlighted. (B1–10) Brightfield images of the arcopallium song nucleus for vocal learners and central intermediate arcopallium (ciA) in non-learners (odd numbers), and the arcopallium area adjacent to the song nucleus or ciA (even numbers). (C1–5) Darkfield images of PV expression in nXIIts and SSp for each species. (D1–10) Brightfield images of SSp (odd numbers) and nXllts (even numbers) for each species. Scale bar in D10 = 30 µm (applies to all brightfield images).

**Figure 4:**
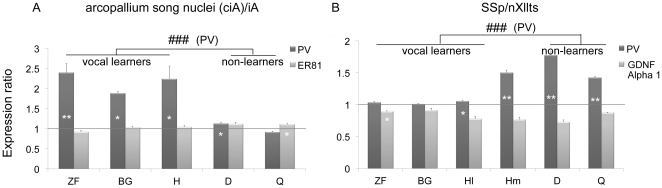
PV mRNA expression ratios measured from the in situ hybridizations in avian species. (A) PV expression ratio of arcopallium song nuclei and adjacent intermediate arcopallium (iA) for vocal learners, and ciA and adjacent intermediate arcopallium (iA) for non-learners. Also quantified is a control gene, ER81. The line across the graph is at ratio = 1, which means both areas equally express the gene. (B) PV expression ratio of SSp and nXllts, and another control gene GDNF family receptor alpha 1. Stars (*) inside bars indicate significant difference between two areas (A. arcopallium song nuclei or ciA and surrounding iA. B. SSp and nXllts) within each species using paired *t*-test on raw values. Number symbol (#) above bars indicates a significant difference between vocal learners and non-learners using unpaired *t*-test on ratios. ZF, zebra finch; BG, budgerigar; H, hummingbird; Hl, humming bird lateral part of nXllts; Hm, hummingbird medial part of nXllts; D, dove; Q, quail. *p<0.05, **p<0.001.

We performed in situ hybridization verifications with zebra finch PV cDNA clones (see methods) from our full-length cDNA collection [46]. The in situ hybridizations confirmed that the arcopallium song nuclei had differentially higher expression relative to the adjacent iA in representative species of all three avian vocal learning lineages (Fig. 3A1–3, 3B1–6). The expression patterns were consistent throughout the nucleus as seen in multiple sections that spanned the song nucleus of each species, and the result in zebra finch was seen with multiple splice variants (not shown). In vocal non-learners, PV expression was sparser and more even throughout the iA (Fig. 3A4–5, 3B7–10). Paired *t*-tests revealed that the differences with the adjacent iA in vocal learners were significant (Fig. 4A, * inside bars). There was no significant expression difference between the ciA and adjacent iA in quails. We found a significant difference in doves, but the magnitude of the difference was much smaller than those in vocal learners (Dove around 0.1; Vocal learners 1.5–2.5) (Fig. 4A, * inside bars).

Since the PV clone used in this study was from zebra finch, the cRNA probe will not hybridize equally across species more distantly related with different levels of sequence homology. To normalize this difference, we ran analyses using ratios of the arcopallium song nuclei and adjacent iA for vocal learners and ciA and adjacent iA for non-learners. This ratio provides an internal control when comparing different species. Unpaired *t*-test on the ratios showed that the vocal learners were significantly different from the vocal non-learners (Fig. 4A, # above bars).

**Figure 5:**
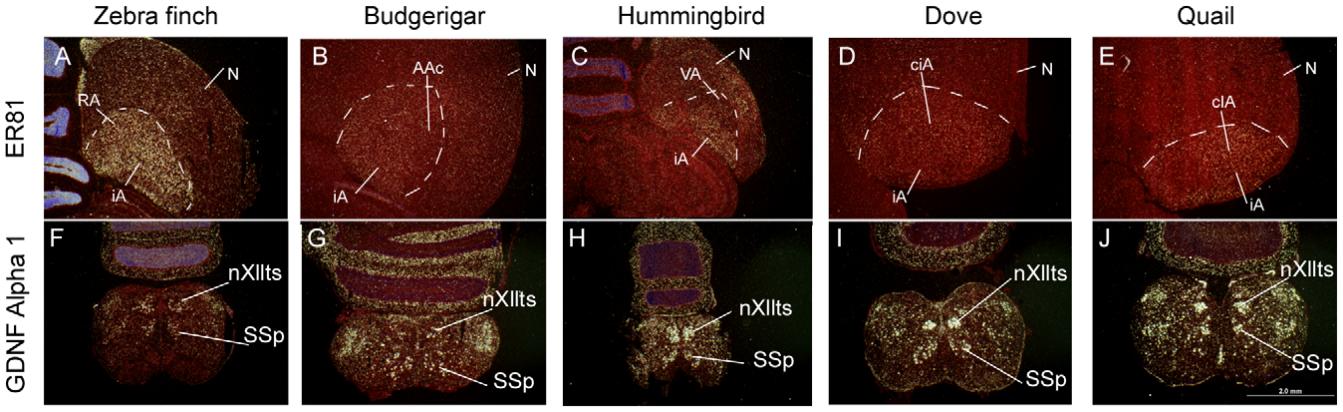
mRNA expression of control genes in avian vocal learners and non-learners. (A–E) ER81, used as an arcopallium control gene. (F–J) GDNF family receptor alpha 1, used as a brainstem motor neuron control gene. Species are labeled above the panels. Scale bar = 2 mm (applies to all the images).

As a control gene we used ER81, which is enriched in the arcopallium of birds and the analogous layer 5 cells of mammalian cortex [41], [47]. Although we found a very small significant difference in quails, there was no large significant differences in the arcopallium song nuclei or ciA versus iA between vocal learners and non-learners for ER81 (Fig. 4A, * inside bars; Fig. 5A–E). Using ratios, we still did not find a significant difference in ER81 between vocal learners and non-learners. Similar to PV, ER81 hybridization was stronger in zebra finches compared to other species, again presumably due to lower homology of the finch probe to the other species (unpublished comparisons to chicken and our parrot genome sequences). On the microarrays ER81 also showed no large differences between vocal learners and non-learners (rank 27,046 of 44,000, unadjusted p = 0.04). These results indicate that the differential expression of PV in the arcopallium song nucleus was specific to PV in all three vocal learning species and not due to other factors, such as cell density. If cell density was the factor responsible for differential expression, ER81 should show a difference as well.

**Figure 6:**
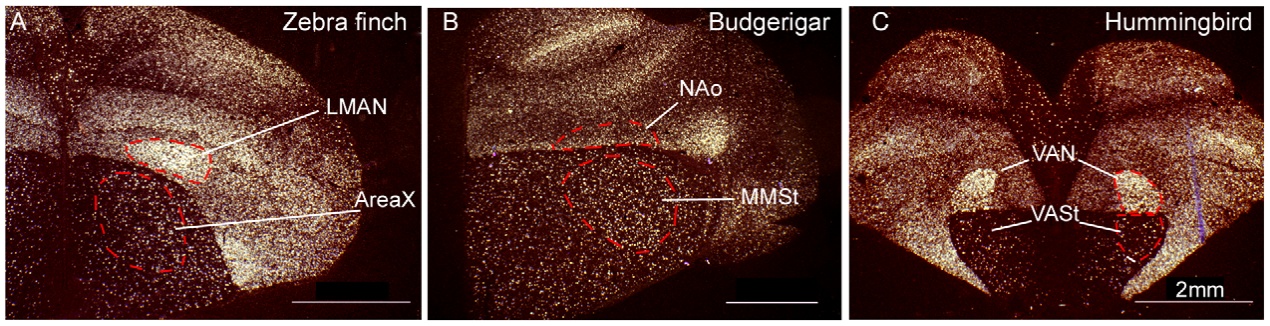
PV mRNA expression in pallial-basal ganglia song pathway nuclei of avian species. (A) PV expression in anterior pathway song nuclei (LMAN and Area X) of zebra finch. (B) PV expression in anterior pathway song nuclei (NAo and MMSt) of budgerigar. (C) PV expression in anterior pathway song nuclei (VAN and VASt) in Anna's hummingbird. Higher PV expression was found in the anterior pathway pallial song nuclei of two species only, zebra finch and Anna's hummingbird. Images are representative of three animals each species. Scale bar = 2 mm (applies to all the images).

In our in situ hybridizations, we visually noted higher PV expression also in the nidopallium song motor pathway nucleus, the HVC analog (zebra finch HVC [no abbreviation]; budgerigar central nucleus of the lateral nidopallium [NLC]; Anna's hummingbird vocal nucleus of the lateral nidopallium [VLN]), relative to the surrounding cells in all three vocal learners (Fig. 3A1–3). This pattern was not observed in the comparable nidopallium regions of vocal non-learners (Fig. 3A4,5 and sections not shown throughout caudal-lateral nidopallium). We also examined PV expression in the anterior pallial-basal-ganglia-thalamic pathway song nuclei and found higher PV expression in the anterior nidopallium song nucleus of two species (zebra finch lateral magnocellular nucleus of the anterior nidopallium [LMAN]; Anna's hummingbird vocal nucleus of the anterior nidopallium [VAN], but not in budgerigar oval nucleus of the anterior nidopallium [NAo]) relative to the surrounding nidopallium (Fig. 6A–C). Again, this pattern was not observed in the comparable nidopallium regions of vocal non-learners (not shown). The striatal song nucleus of vocal learners (zebra finch Area X [no abbreviation]; budgerigar magnocellular nucleus of the medial striatum [MMSt]; and Anna's hummingbird vocal nucleus of anterior striatum [VASt]) did not show notable differential expression relative to the surrounding striatum for any species (Fig. 6A–C). In summary, these data suggest that there has been convergent up-regulation of PV expression in the posterior song motor pathway of all three avian vocal learning lineages, and convergence in one nucleus of the song pallial-basal-ganglia pathway in two of the lineages.

### PV mRNA expression is specialized in the brainstem vocal nucleus of avian vocal learners

We next examined PV expression in the brainstem. Since the vocal (nXIIts) and neck (SSp) motor neurons are homologous among vocal learners and non-learners and are derived from the same somatic embryonic motor neuron pool [20], it is traditionally thought there would unlikely be molecular differences between vocal learning and non-learning species. However, we surmised that there might be differences between SSp and nXllts, since SSp does not receive a direct projection from the forebrain (from iA), whereas nXllts does (from the RA analogs). We found differences in PV expression in the microarrays for the finches and budgerigars (Fig. 2B). We verified the microarray results by in situ hybridization, and found high PV mRNA expression in nXIIts of zebra finches and budgerigars (Fig. 3C1,2; 3D1–4), but barely detectable expression in nXIIts of quail and doves (Fig. 3C4,5; 3D7–10). Interestingly, in the Anna's hummingbird, the medial part of nXIIts showed low PV expression, but the lateral part consistently showed isolated cells with high expression (Fig. 3C3, 3D6), which appears to have influenced the microarray result of overall lower expression. Quantitative analysis with paired *t*-tests between nXllts and SSp of each species confirmed the differential expression, including differences in the medial and lateral parts of Anna's hummingbird nXllts (Fig. 4B, * inside bars). When we compared ratios of SSp to nXllts expression (lateral part for hummingbird), unpaired t-test showed that the vocal learners were significantly different from the vocal non-learners (Fig. 4B, # above bars). The high PV mRNA expression in vocal learners was specific to the very large cells (Fig. 3D), that is the motor neurons. No differences between vocal learners and non-learners were found in SSp and nXllts for the expression of a control gene, GDNF family receptor alpha 1, which we found to be a motor neuron marker (Fig. 4B; Fig. 5F–J). Rather, there was slightly less GDNF family receptor alpha 1 expression in nXIIts relative to SSp across all species tested, vocal learners and non-learners. These findings indicate that the difference of PV expression in the brainstem vocal motor nucleus of vocal learners versus non-learners is not due to differences in overall gene expression. The differences are due specifically to PV expression levels.

In summary, these data suggest one of two possibilities: 1) convergent up-regulation of PV expression in the brainstem vocal motor neurons of all three avian vocal learner lineages; or 2) convergent down-regulation of PV in vocal non-learners. The low nXIIts expression relative to SSp only in vocal non-learners is counterintuitive to our expectations, as it indicates that the nXIIts in vocal non-learners maybe the nucleus with specialized expression.

### PV is also expressed at high levels in the human nXII motor neurons

In mammals, the functional analog of avian nXIIts is Amb, which innervates muscles of the larynx. However, the mammalian anatomical homolog of avian nXll (both caudal ts and rostral tongue parts) is nXII, which innervates the mammalian tongue (Fig. 1B [6], [48]). We did not note a difference of PV expression in the rostral and caudal nXII in songbirds (not shown). Among primates, nXII receives a strong direct projection from the face motor cortex in humans, a moderate direct projection in chimpanzees, and weak one in macaques [6], [14], [15], [18]. PV protein is known to be expressed at low levels relative to other motor neurons in Amb of mammals, including in non-human primates [49]. We are not aware of any studies that have tested PV mRNA expression in human nucleus Amb and nXII. Thus, we obtained post-mortem human brain samples of normal donors through the Kathleen Price Bryan Brain Bank at Duke University and fresh frozen Rhesus macaque (*Macaca mulata*) brains from the Oregon National Primate Research Center, and processed brainstem sections for PV expression. We also processed adjacent sections for expression of GDNF family receptor alpha 1 to help identify the locations of nXII and Amb motor neurons.

**Figure 7:**
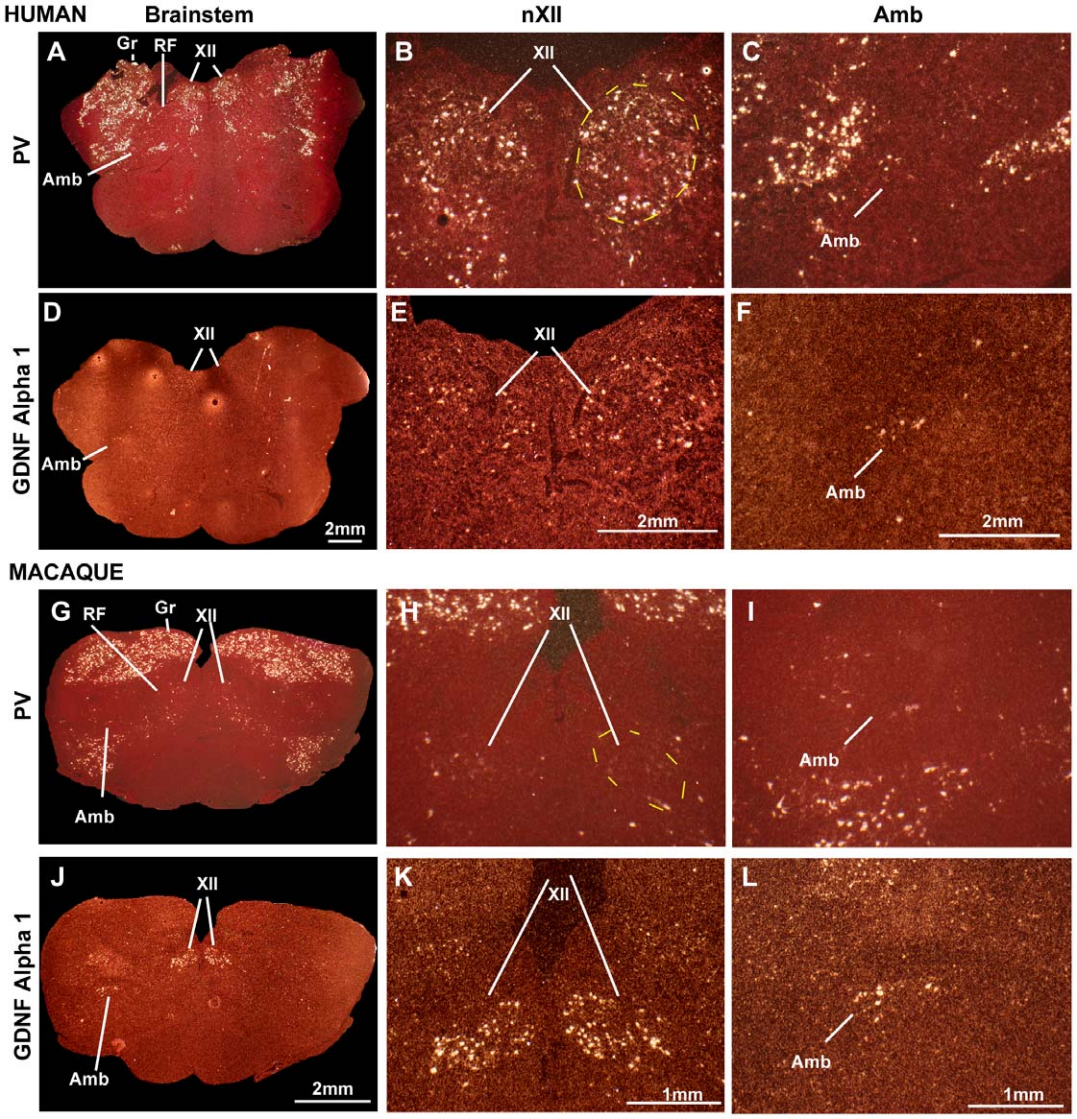
PV and GDNF family receptor alpha 1 mRNA expression in human and macaque brainstem. (A–C) PV expression in human, including human nXll, Amb, and sensory nuclei; (D–F) GDNF family receptor alpha 1 expression in human identifying nXII and Amb motor neuron populations. (G–I) PV expression in macaque showing the homologous brain regions; (J–L) GDNF family receptor alpha 1 expression in macaque to identify the motor neuron populations. The first column (A, D, G, J) shows low magnification of the entire brainstem section, the second column (B, E, H, K) shows the location of nXII and the third (C, F, I, L) shows the location of Amb. Note the high PV expression in human nXII and low in macaque nXII, but more comparable expression of GDNF family receptor alpha 1 in both species. Scale bar = 2 mm (applied to D, E, F, J) and 1 mm (applied to K, L).

Similar to vocal learning versus vocal non-learning birds, we found significantly higher PV expression in the nXII motor neurons in humans (Fig. 7A,B) relative to macaques (Fig. 7G,H). We were able to locate nXII and Amb in adjacent sections using the GDNF family receptor alpha 1 label (Fig. 7D–F, 7J–L), but for Amb, appropriate for its name, the number of motor neurons in each section were few and PV expression in this region was scattered, making it difficult to determine whether labeled PV neurons belonged to Amb or to adjacent regions (Fig. 7C,F,I,L). Thus, we focused further quantitative analyses on nXII. We sought a means to quantify the labeled cells in nXII, as SSp is not in the same plane of section in the primate brains, and we noted that unlike vocal non-learning birds, macaques did have isolated cells with high PV expression, particularly at the lateral edge of the nucleus (Fig. 7H). We also noted that the sensory neuron populations in the human and macaque brainstem, like in birds, had high levels of PV expression. Thus, we normalized our quantification to the somatosensory nuclei (e.g. gracile [Gr]; see methods).

**Figure 8:**
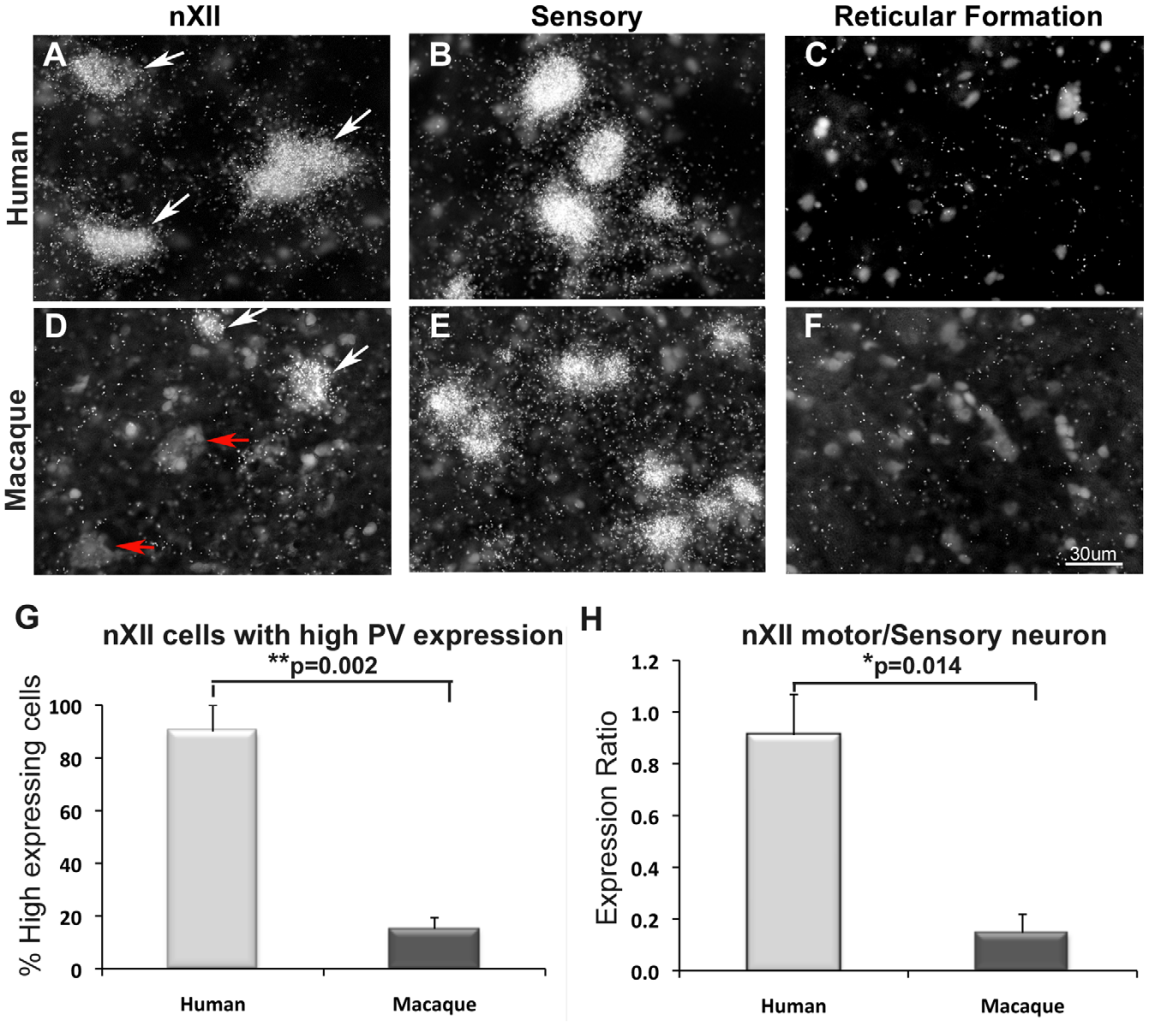
High power view and quantification of PV in nXII motor neurons of human and macaque. (A–F) Darkfield view (inverted from brightfield) of labeled (white arrows; silver-white grains over cell bodies) and non-labeled (red arrow) cells in nXII, sensory nuclei (Gr), and adjacent reticular (RT) formation of human (A–C) and macaque (D–F). Images are from the same human (A–C) and macaque (D–F) individuals for accurate comparison, but are representative of all individuals. Many cells with high PV expression were observed in human nXII and sensory nuclei, but in macaque only few were seen in nXII, mainly at the periphery of the nucleus. (G) Percent of XII motor neurons with PV expression levels at ∼25% of or greater than seen in the sensory neurons of the same individual human or macaque. Motor neurons were recognized by their large size. (H) Ratio of the PV expression (% area of silver grains over cell bodies) of the XII motor neurons divided by the average of the sensory neurons for each individual human and macaque. * p-values are from unpaired t-test. Scale bar = 30 µm (applies to all the images).

We found that ∼90% of the cells in human nXII expressed high PV levels (∼25% of or greater than in the sensory neurons), whereas only ∼15% did so in macaques (Fig. 8A,D,G). A ratio analysis revealed that humans had significantly higher average PV expression levels per nXII motor neuron relative to sensory neurons (ratio ∼0.9), whereas macaques had a significantly lower relative levels (ratio ∼0.14) (Fig. 8A,B, D,E,H). There was very little PV expression in the reticular formation ventral to the sensory neurons (Fig. 8C,F), further supporting the finding that the difference between human and macaque does not reflect an overall expression difference in the brain sections. Rather, these findings show that there is a large and significant difference of PV expression in human versus macaque nXII motor neurons, both in the proportion of highly labeled cells and in the amount of label per cell. They further indicate that in parallel with known differences in neural connectivity, humans share high differential expression of PV in nXII motor neurons with vocal learning birds, macaques have intermediate levels, and vocal non-learning birds have the lowest levels.

## Discussion

### PV may play an important role in multiple independent lineages of vocal learners

Prior studies in songbirds reported higher PV protein and mRNA expression in RA and HVC relative to the surrounding brain regions [22], [37], [50], but no comparisons were made with other vocal learning as well as vocal non-learning lineages. In the budgerigar, other brain areas were studied using immunohistochemistry for PV, but PV distribution in song nuclei was not determined [51]. Thus, it was not known if this specialized PV expression is specific to songbirds or convergent among vocal learners. Our study found that PV expression was selectively higher in the RA and HVC analogs as well as in the brainstem vocal motor neurons in all three avian vocal learning lineages. Further, we found that the homologous brainstem tongue motor neurons in humans had higher PV expression relative to macaques. We were not able to assess PV expression in the oral facial motor cortex of humans and non-human primates, the regions considered analogous to avian HVC and RA analogs [2], but interestingly, Sherwood et al 2004 [52] showed that PV-ir interneurons are proportionally more frequent in the orofacial primary motor cortex (i.e. face motor cortex) in hominids (humans and great apes) compared with macaques, whereas visual cortex does not show this relative increase [53]. Moreover, the hominid orofacial cortex and songbird RA are both unusual in that in addition to their GABAergic neurons, their projection neurons (layer 5 in hominids and RA to nXIIts projecting neurons in songbirds) also express PV (although weaker than in GABAergic neurons) [22], [52], a finding rarely seen in the mammalian brain [54]. In contrast, female zebra finches and macaques, which do not learn vocalizations appear to not have such PV positive projection neurons in their arcopallium or orofacial motor cortex, respectively [22], [52], although an earlier study found them in other parts of macaque motor cortex [55]. The authors of both the human and songbird studies [22], [52] independently suggested that the PV specialization in the projection neurons could be related to the evolution of vocal and orofacial mimicry. Combined with our findings, we suggest that there might be convergent PV up-regulation in forebrain and brainstem areas among vocal learners that span over 300 million years from a common ancestor [41].

Our finding is the only one that we are aware of showing convergent differential regulation of a gene in distantly related vocal learning birds and in humans. Other studies have examined only one or two lineages, only birds, or only mammals, identifying differences in neurotransmitter receptors, cell adhesion molecules and transcription factors among several vocal learning avian species [23], [28], [29], [32], [56], and the FoxP1 transcription factor across all three avian vocal learners [19], [24], [25]. Thus, further study is necessary to determine if there are any other genes that show the same level of expression convergence as PV, which we will be able to discover from our microarray experiments. The most parsimonious interpretation of our findings is that up-regulation of PV expression in motor regions for song and speech has been selected for each time the vocal learning trait evolved. Future studies will be necessary to determine if this is true for other mammalian vocal learners, such as bats, dolphins, pinnepeds, and elephants [1], [2], [57]. Nevertheless, the discovered association thus far suggests an important, unexpected, enhanced role of PV in learned vocal communication.

The main physiological role of PV in the brain is to buffer calcium. Two consequences of this buffering have been proposed: (1) the common view of neuroprotection against calcium toxicity induced by high levels of neural activity, such as that seen in fast spiking GABAergic inhibitory interneurons; and (2) a less common view for neural plasticity, by modulating Ca2+ signaling pathways and critical periods also in GABAergic interneurons [35], [36]. Consistent with the first hypothesis, motor nuclei in the brainstem and spinal cord that contain high PV levels show much less calcium deposits (an indicator of neuron damage) than those that do not contain PV [58]. Experimental over-expression of PV in motor neurons that normally express low levels of PV reduces the formation of calcium deposits and axotomy-induced death to a degree comparable with motor neurons that normally express high levels of PV [59]. Similar to PV expression, song motor pathway nuclei in songbirds have higher levels of cytochrome oxidase activity indicative of higher metabolic rates relative to the surrounding brain subdivisions [60], suggesting that they may need extra neuroprotection. Consistent with the second hypothesis, the number of PV-positive GABAergic interneurons surrounded by perineuronal nets (PNN) increases in the mammalian visual cortex and songbird HVC (GABAergic status not known) during the critical periods for ocular dominance and song learning, respectively [37], [61]. For ocular dominance column formation, when the PNN or GABAergic neurons are inactivated in the visual cortex, the critical period is extended [61]. For song learning, song production variance (measured as entropy variance and frequency variance) positively correlates with the presence of PV labeled PNN neurons in HVC [37]. Non-PNN PV-positive neurons were not assessed.

The above findings suggest that both hypotheses could be correct. Specifically, we hypothesize that enhanced expression of PV in vocal motor pathways may have been selected for to either enhance neuroprotection and plasticity of vocal production and vocal learning pathways, relative to other behaviors, multiple independent times. Enhanced protection may allow vocal learners to vocalize more often than vocal non-learners. Enhanced plasticity might allow vocal learners to have more flexible vocal behavior than other behaviors. These hypotheses are testable and should lead to greater insight into what makes song and speech special in vocal learning species.

## Materials and Methods

### Subjects

We used male zebra finches (*Taeniopygia guttata* n = 6), adult male budgerigars (*Melopsittacus undulatus* n = 4), male Anna's hummingbirds (*Calypte anna* n = 3), male ring doves (*Stereptopelia capicola* n = 4), and adult male Japanese quails (*Coturnix japonica* n = 3). All species, except hummingbirds, were bred in our aviaries at Duke University. The hummingbirds were captured in Riverside, California [19]. All of our animal experiments were performed according to Duke University guidelines and approved by Duke University Animal Care and Use Committee (protocol number: A107-08-04). We chose males for avian species, as they are usually the vocal learning sex, and males for all species to eliminate potential confounds of sex differences. Fresh frozen Rhesus macaque (*Macaca mulata; n = 3*) brainstem samples of males, age 8–9 years old, were obtained from the Oregon Primate Center. Some of the sections were cut at the Duke Histology laboratory. Human brainstem samples (n = 3), two males and one female, and over 80 years old, were obtained from the Kathleen Price Bryan Brain Bank at Duke University. A Standard Operating Procedure for handling human and non-human primate tissues was approved by the Duke University Occupational Safety Office.

### Behavior

All the avian species, except hummingbirds were isolated in sound attenuation boxes overnight. The box light was turned on the next morning for 1 hour and behavior was monitored through a camera inside of the box. Birds were provided with food and water. We used animals that did not sing in the morning, because we needed to avoid identifying neural activity-induced genes that are regulated in song nuclei by singing [46], [62]. Hummingbirds were captured in the wild using sugar water bottle traps. Prior to capture, we observed their behavior for about 1 hour after dawn using binoculars. For this experiment, we used those did not yet sing. For macaques and humans, all subjects were reported as cognitively normal, but we are not aware of the macaque's and person's vocalization status before death.

### Tissue preparation

After the behavioral observations, birds were sacrificed by quick decapitation. Brains were quickly removed (within 5 min) and embedded in OCT compound (Sakura Fine Technical, Japan) in a plastic block mold, frozen in a dry ice-ethanol bath, and kept at −80°C until use. Macaque samples were obtained within 10–15 minutes of sacrifice, the medulla was dissected, frozen on dry ice, and shipped to our lab. Human samples that gave detectable PV signal in the in-situ hybridizations were those obtained within 6–17 hrs post mortem; those obtained after 20 hrs post mortem did not have reliable signal. From these samples, the medulla was dissected, and frozen in OCT in a block mold. Coronal, frozen brains were cut at 10 µm thickness for birds and 12 um for human and macaques. Some sections were saved on polyethylene naphthalate membrane (PEN) slides (Molecular Devices, USA) for laser capture and others were mounted on plus charge slides (Fisher Scientific, USA) for in situ hybridizations. Slides were stored at −80°C until processing.

### Laser captured microdissection (LCM), RNA isolation and cDNA synthesis

PEN membrane slides were removed from −80°C and placed in fresh 75% ethanol diluted with sterile RNAse free distilled water for 5 min in an RNAse free designated hood. Slides were rinsed in distilled RNAse free water until OCT compound was dissolved. For brainstem sections, we stained with 0.3% cresyl violet in RNAse free water for 5 min to visualize nuclei better. For the forebrain sections, we did not stain them since song nuclei were easily seen due to their increased fiber density. The slides were then dehydrated in a series of freshly prepared alcohols, several dips each of 75%, 95% and 100%, in sterile 50 ml tubes with 2 changes each. The slides were placed in fresh xylene twice for 5 min each and then placed in the hood until the tissue dried (∼5 min or more). Slides were then placed under an Arcturus XT laser microdissection microscope (Molecular Devices). Target areas (RA-analog song nucleus for vocal learners, ciA for non-learners, medial or ventral-lateral iA depending on species, nXIIts, and SSp) were identified and manually outlined with the software drawing tool, adhered to Capsure Macro LCM caps (Molecular Devices) with an IR laser, cut with the UV laser, and then captured to the cap. We captured an average of 5 sections for arcopallium regions and 10 for brainstem nuclei per cap. After capture, the cap membrane with nuclei were carefully removed and placed in a 0.65 ml tube of the PicoPure RNA isolation kit which contained 50 ul of disruption buffer (Molecular Devices). Tubes were placed on a 42°C heat block for 30 min then in a −80°C freezer until all desired samples were captured. Total RNA was then isolated according to the remaining protocol steps in the PicoPure isolation kit instructions (Molecular Devices). The concentration and integrity of total RNA were measured on a 2100 Bioanalyzer (Agilent Technologies, USA) using the RNA Pico 6000 kit according to manufacturer instructions (Agilent Technologies). Five nanograms of total RNA were linearly amplified as cDNA using the uMACS SuperAmp Kit (Miltenyi Biotec, Germany). Just before cDNA amplification, a 1∶200000 dilution of Agilent One Color RNA Spike-In Mix (Agilent Technologies) was added to five nanograms of total RNA from each sample. The Spike-in recognizes only control non-vertebrate oligos on the array, which allows us to detect amplification and hybridization artifacts. Samples were linearly amplified as cDNA and labeled with Cy3-dCTP using the uMACS SuperAmp Kit (Miltenyi Biotec). After completing the reaction, the synthesized Cy3 labeled cDNA concentration was calculated with a NanoDrop 2100 (Thermo Scientific, USA). For this experiment, we used three animals for each bird species.

### Microarray hybridization and analyses

From the amplified cDNA reactions, 1.5 mg of amplified labeled product (probe) was denatured and hybridized to our custom designed songbird oligo spotted arrays (Agilent Technologies Songbird Array v2 [44]; Whitney, et al. submitted) containing oligos designed from over 44,000 relatively unique cDNA transcripts, including some splice variants. More detailed information on the arrays is available at http://aviangenomes.org/main/zebrafincholigoarray. For hybridization, the Maui hybridization system was used (BioMicrosystems, USA). The arrays were hybridized at 55°C for zebra finch probes. For the other bird species, we found that we needed to hybridize them at a lower temperature, 46°C, to obtain comparable detectable signals presumably due to lower sequence homologies.

After hybridization, the microarrays were scanned with the Axon GenePix 4000B scanner to acquire and analyze the expression data (Molecular Devices). For analysis, signal intensity on an axon array scanner was obtained in an Agilent oligoarray format. The raw data has been deposited in a MIAME compliant database, GEO (accession # GSE28395 for the arcopallium experiments and GSE33667 for the brainstem experiments). The data was extracted in R using the Agi4×44PreProcess Bioconductor library (R Foundation for Statistical Computing, Austria). The values were normalized using median centered log2 transformation. Raw and normalized expression distributions were evaluated for sample quality control using the normalization centering profile, the normalization factor, and a cross-sample correlation analysis. Normalization was evaluated with VSN (variance stabilization normalization)-Scale Factor package in R. VSN-Scale Factor was chosen because it performed the least manipulation of the original intensity profiles, and normalizes samples among themselves. Because avian species more distant from zebra finch may not efficiently produce a signal from all oligos, we normalized based on a scale factor within species and then compared results across species. Scale Factor normalizes based upon the 40^th^ to 60^th^ percentile range of detected oligos, and produced output suitable to review the detection efficiencies across all oligos. When a sample's normalization factor exceeded 10 or when the normalization centering profile substantially diverged from those of the same species, microarray hybridization of the sample was repeated. One of the quail samples was discarded due to low quality hybridization, even upon replication.

Due to the noisy nature of microarrays, which is further enhanced by cross-species hybridizations, we decided to perform microarray statistical analyses on traits (vocal learning vs non-learning) as the grouping variable rather than species. This approach does not allow independent analyses within species, but increases sample size of the group of interest. To perform a sensitive test of how much a gene differs between brain regions relative to all other genes on the array, normalized data was statistically analyzed using the Bioconductor R package using the empirical Bayes (eBayes) method of the “limma” to adjust the t-statistics as described for microarays [63] (http://rss.acs.unt.edu/Rdoc/library/limma/html/ebayes.html). First, the log2 normalized intensities of the experimental region (e.g. RA or ciA) were subtracted from the control region (iA) in per pairwise comparisons for each bird, yielding residual log2 intensities. We extracted the matrix data for only the subset of relevant samples for comparison, where each sample (animal) was a row, and the columns represent vocal learners (n = 9; 3 individuals ×3 species) and vocal non-learners (n = 5; 3 doves+2 quails). Then we fitted a linear model (using lmFit, function in R) using the subset matrix, and the design matrix. We then computed a moderated *t*-statistic using an empirical Bayes shrinkage of standard errors (using the eBayes, function in R). We generated summary tables using the topTable function in R. These files were created as text files with the file name containing “pairedRatiosVocalTtest”. This analyses yielded ranked list of transcripts that were differentially expressed in vocal learners versus non-learners at p<0.01. The topTable was used to generate Benjamini-Hochberg false discovery rates (FDR), commonly used in microarray analyses, at p<0.2. This FDR method provided a good balance between discovery of statistically significant genes and limitation of false positive occurrences (http://www.silicongenetics.com/Support/GeneSpring/GSnotes/analysis_guides/mtc.pdf). However, in practice, we did not use the values as it was not informative for identifying true positives.

### In situ hybridization

Radioactive ^35^S in situ hybridizations were performed as previously described [23]. In brief, sections were fixed in 3% paraformaldehyde, rinsed with 1× phosphate-buffer saline (PBS), dehydrated in ethanol, air dried, and hybridized with 1×10^6^ cpm per slide of antisense and sense ^35^S-UTP-labeled riboprobes of the gene of interest. We generated riboprobes from cloned cDNA of zebra finch PV (NCBI accession # DQ215755), zebra finch ER81 (accession # DV582566), chicken GDNF family receptor alpha1 (accession # NM_205102), human. PV (accession # NM_145793.3), and GDNF family receptor alpha 1 (accession # NM_002854). Clones were from our cDNA library collection [46], except human PV and GDNF family receptor alpha 1, which were from Thermo Scientific. We used some slides from the same animals that we used for microarray experiments, as well as additional animals to confirm the patterns. We hybridized at 65°C for zebra finch, human, and macaque. We used a lower hybridization temperature, 60°C, for species of other avian orders. After hybridization, slides were dehydrated and exposed to X-ray films (Kodak, USA) for 1–7 days. Slides were then dipped in autoradiographic emulsion (Kodak) and incubated at 4°C for 1–2 days for zebra finch PV and 3 days for the other species. They were then processed with D-19 developer (Kodak) and fixer (Kodak), washed, counterstained with cresyl-violet acetate solution (Sigma, USA) and coverslipped with Permount (Sigma). Sense probes did not show any specific signals.

### Quantification and Statistics for in situ hybridization

Quantification of in situ images for birds was performed similarly as previously described [46]. Autoradiographic images of brain sections exposed to X-ray films were digitally captured using an Olympus MVX10 microscope (Olympus, Japan) connected to a DP71 camera (Olympus) and DP Controller software. Adobe Photoshop CS3 (Adobe Systems, USA) was used to measure the mean pixel intensities on a 256 gray scale in the areas of interest. We measured two adjacent areas and used the average for statistical analyses. We did not subtract the background value on the slide without tissue, because some regions (i.e. PV in non-learner nXIIts) showed barely any expression, sometimes resulting in negative values. Further, our ratio analyses reduced the need for background values. All the background values did not significantly differ from each group and each animal. First, we used paired t-tests on the raw values to test for significant differences within each species. Second, we used ratios and unpaired t-tests to test for differences between vocal learning and non-learning groups.

A quantification for human and macaque sections was done differently due to: 1) greater spacing of motor neurons in these larger brains, making it difficult to quantify from the X-ray films; and 2) variation in signal intensity of the human samples as a result of variation in time of freezing the tissue post mortem. We took brightfield images of nXII, sensory nuclei (e.g. Gr) and other brain regions using a compound microscope (Olympus) at 40X magnification from at least 2 adjacent sections. We then used the threshold function of Image J from NIH (Wayne Rasband) to select as many silver grains as possible without selecting the Nissl stain signal. We then used the drawing selection tool to draw an outline around each motor or sensory neuron, and then measured the % area taken up by selected thresholded grains for 10–20 neurons for each individual per brain region. The % area taken up by silver grains was divided by the area of the cell body selected. We then subtracted out background from label in the reticular formation cells or equivalent size area in the neuropil between motor neurons to obtain a final number of % area-background. The background grains typically ranged from 0.5–4%. To calculate the relative number of cells in XII that had high expression levels of PV, we used a cut off of ∼25% of or greater than the level seen in the sensory neurons of the same individual human or macaque, from the same brain sections. The value of 25% or greater approximately corresponded to what we perceive by eye as highly labeled (in Fig. 8A–F, arrows). To calculate the ratio of the PV expression in nXII versus sensory neurons, we used % area average of the XII motor neuron cells divided by the % area average of the sensory neurons for each individual human and macaque. This is similar to the expression ratio calculation between nXIIts and SSp in birds, except using the sensory neurons in the same section instead of SSp. Statistical analyses between human and macaque were conducted using unpaired *t*-test.

## References

[1] Janik VM, Salater PB (1997). Vocal learning in mammals.. Adv Study Behav.

[2] Jarvis ED (2004). Learned birdsong and the neurobiology of human language.. Ann N Y Acad Sci.

[3] Doupe AJ, Kuhl PK (1999). Birdsong and human speech: common themes and mechanisms.. Annu Rev Neurosci.

[4] Hauser MD, Chomsky N, Fitch WT (2002). The faculty of language: what is it, who has it, and how did it evolve?. Science.

[5] Nottebohm F (1972). The origins of vocal learning.. The American Naturalist.

[6] Jurgens U (2002). Neural pathways underlying vocal control.. Neurosci Biobehav Rev.

[7] Fitch WT, Huber L, Bugnyar T (2010). Social cognition and the evolution of language: constructing cognitive phylogenies.. Neuron.

[8] Nottebohm F, Stokes TM, Leonard CM (1976). Central control of song in the canary, Serinus canarius.. J Comp Neurol.

[9] Scharff C, Nottebohm F (1991). A comparative study of the behavioral deficits following lesions of various parts of the zebra finch song system: implications for vocal learning.. J Neurosci.

[10] Jarvis ED, Ribeiro S, da Silva ML, Ventura D, Vielliard J (2000). Behaviourally driven gene expression reveals song nuclei in hummingbird brain.. Nature.

[11] Wild JM (1997). Neural pathways for the control of birdsong production.. Journal of Neurobiology.

[12] Deacon TW (2000). Evolutionary perspectives on language and brain plasticity.. J Commun Disord.

[13] Fischer J, Hammerschmidt K (2010). Ultrasonic vocalizations in mouse models for speech and socio-cognitive disorders: insights into the evolution of vocal communication.. Genes Brain Behav.

[14] Kuypers HG (1958). Some projections from the peri-central cortex to the pons and lower brain stem in monkey and chimpanzee.. J Comp Neurol.

[15] Kuypers HG (1958). Corticobular connexions to the pons and lower brain-stem in man: an anatomical study.. Brain.

[16] Wild JM (1994). The auditory-vocal-respiratory axis in birds.. Brain Behav Evol.

[17] Wild JM, Li D, Eagleton C (1997). Projections of the dorsomedial nucleus of the intercollicular complex (DM) in relation to respiratory-vocal nuclei in the brainstem of pigeon (Columba livia) and zebra finch (Taeniopygia guttata).. J Comp Neurol.

[18] Jurgens U, Alipour M (2002). A comparative study on the cortico-hypoglossal connections in primates, using biotin dextranamine.. Neurosci Lett.

[19] Feenders G, Liedvogel M, Rivas M, Zapka M, Horita H (2008). Molecular mapping of movement-associated areas in the avian brain: a motor theory for vocal learning origin.. PLoS One.

[20] Dubbeldam JL (1998). The neural substrate for ‘learned’ and ‘nonlearned’ activities in birds: a discussion of the organization of bulbar reticular premotor systems with side-lights on the mammalian situation.. Acta Anat (Basel).

[21] Bottjer SW, Brady JD, Cribbs B (2000). Connections of a motor cortical region in zebra finches: relation to pathways for vocal learning.. J Comp Neurol.

[22] Wild JM, Williams MN, Suthers RA (2001). Parvalbumin-positive projection neurons characterise the vocal premotor pathway in male, but not female, zebra finches.. Brain Res.

[23] Wada K, Sakaguchi H, Jarvis ED, Hagiwara M (2004). Differential expression of glutamate receptors in avian neural pathways for learned vocalization.. J Comp Neurol.

[24] Haesler S, Wada K, Nshdejan A, Morrisey EE, Lints T (2004). FoxP2 expression in avian vocal learners and non-learners.. J Neurosci.

[25] Teramitsu I, Kudo LC, London SE, Geschwind DH, White SA (2004). Parallel FoxP1 and FoxP2 expression in songbird and human brain predicts functional interaction.. J Neurosci.

[26] Li X, Wang XJ, Tannenhauser J, Podell S, Mukherjee P (2007). Genomic resources for songbird research and their use in characterizing gene expression during brain development.. Proc Natl Acad Sci U S A.

[27] Lovell PV, Clayton DF, Replogle KL, Mello CV (2008). Birdsong “transcriptomics”: neurochemical specializations of the oscine song system.. PLoS One.

[28] Matsunaga E, Kato M, Okanoya K (2008). Comparative analysis of gene expressions among avian brains: a molecular approach to the evolution of vocal learning.. Brain Res Bull.

[29] Matsunaga E, Okanoya K (2009). Evolution and diversity in avian vocal system: an Evo-Devo model from the morphological and behavioral perspectives.. Dev Growth Differ.

[30] Fisher SE, Marcus GF (2006). The eloquent ape: genes, brains and the evolution of language.. Nat Rev Genet.

[31] Johnson MB, Kawasawa YI, Mason CE, Krsnik Z, Coppola G (2009). Functional and evolutionary insights into human brain development through global transcriptome analysis.. Neuron.

[32] Haesler S, Rochefort C, Georgi B, Licznerski P, Osten P (2007). Incomplete and inaccurate vocal imitation after knockdown of FoxP2 in songbird basal ganglia nucleus Area X.. PLoS Biol.

[33] Fisher SE, Scharff C (2009). FOXP2 as a molecular window into speech and language.. Trends Genet.

[34] Horn D, Kapeller J, Rivera-Brugues N, Moog U, Lorenz-Depiereux B (2010). Identification of FOXP1 deletions in three unrelated patients with mental retardation and significant speech and language deficits.. Hum Mutat.

[35] Schwaller B, Meyer M, Schiffmann S (2002). ‘New’ functions for ‘old’ proteins: the role of the calcium-binding proteins calbindin D-28k, calretinin and parvalbumin, in cerebellar physiology. Studies with knockout mice.. Cerebellum.

[36] Hensch TK (2005). Critical period plasticity in local cortical circuits.. Nat Rev Neurosci.

[37] Balmer TS, Carels VM, Frisch JL, Nick TA (2009). Modulation of perineuronal nets and parvalbumin with developmental song learning.. J Neurosci.

[38] Striedter GF (1994). The vocal control pathways in budgerigars differ from those in songbirds.. J Comp Neurol.

[39] Durand SE, Heaton JT, Amateau SK, Brauth SE (1997). Vocal control pathways through the anterior forebrain of a parrot (Melopsittacus undulatus).. J Comp Neurol.

[40] Gahr M (2000). Neural song control system of hummingbirds: comparison to swifts, vocal learning (Songbirds) and nonlearning (Suboscines) passerines, and vocal learning (Budgerigars) and nonlearning (Dove, owl, gull, quail, chicken) nonpasserines.. J Comp Neurol.

[41] Jarvis ED, Gunturkun O, Bruce L, Csillag A, Karten H (2005). Avian brains and a new understanding of vertebrate brain evolution.. Nat Rev Neurosci.

[42] Reiner A, Perkel DJ, Bruce LL, Butler AB, Csillag A (2004). Revised nomenclature for avian telencephalon and some related brainstem nuclei.. J Comp Neurol.

[43] Wild JM, Williams MN, Suthers RA (2000). Neural pathways for bilateral vocal control in songbirds.. J Comp Neurol.

[44] Warren WC, Clayton DF, Ellegren H, Arnold AP, Hillier LW (2010). The genome of a songbird.. Nature.

[45] Roulhac PL, Ward JM, Thompson JW, Soderblom EJ, Silva M (2011). Microproteomics: quantitative proteomic profiling of small numbers of laser-captured cells.. Cold Spring Harb Protoc.

[46] Wada K, Howard JT, McConnell P, Whitney O, Lints T (2006). A molecular neuroethological approach for identifying and characterizing a cascade of behaviorally regulated genes.. Proc Natl Acad Sci U S A.

[47] Nomura T, Takahashi M, Hara Y, Osumi N (2008). Patterns of neurogenesis and amplitude of Reelin expression are essential for making a mammalian-type cortex.. PLoS One.

[48] Roberts TF, Wild JM, Kubke MF, Mooney R (2007). Homogeneity of intrinsic properties of sexually dimorphic vocal motoneurons in male and female zebra finches.. J Comp Neurol.

[49] Reiner A, Medina L, Figueredo-Cardenas G, Anfinson S (1995). Brainstem motoneuron pools that are selectively resistant in amyotrophic lateral sclerosis are preferentially enriched in parvalbumin: evidence from monkey brainstem for a calcium-mediated mechanism in sporadic ALS.. Exp Neurol.

[50] Wild JM, Williams MN, Howie GJ, Mooney R (2005). Calcium-binding proteins define interneurons in HVC of the zebra finch (Taeniopygia guttata).. J Comp Neurol.

[51] Roberts TF, Hall WS, Brauth SE (2002). Organization of the avian basal forebrain: chemical anatomy in the parrot (Melopsittacus undulatus).. J Comp Neurol.

[52] Sherwood CC, Holloway RL, Erwin JM, Hof PR (2004). Cortical orofacial motor representation in Old World monkeys, great apes, and humans. II. Stereologic analysis of chemoarchitecture.. Brain Behav Evol.

[53] Sherwood CC, Raghanti MA, Stimpson CD, Bonar CJ, de Sousa AA (2007). Scaling of inhibitory interneurons in areas v1 and v2 of anthropoid primates as revealed by calcium-binding protein immunohistochemistry.. Brain Behav Evol.

[54] Hof PR, Glezer II, Conde F, Flagg RA, Rubin MB (1999). Cellular distribution of the calcium-binding proteins parvalbumin, calbindin, and calretinin in the neocortex of mammals: phylogenetic and developmental patterns.. J Chem Neuroanat.

[55] Preuss TM, Kaas JH (1996). Parvalbumin-like immunoreactivity of layer V pyramidal cells in the motor and somatosensory cortex of adult primates.. Brain Res.

[56] Ball GF (1994). Neurochemical specializations associated with vocal learning and production in songbirds and budgerigars.. Brain Behav Evol.

[57] Tyack PL (2008). Convergence of calls as animals form social bonds, active compensation for noisy communication channels, and the evolution of vocal learning in mammals.. J Comp Psychol.

[58] Obal I, Engelhardt JI, Siklos L (2006). Axotomy induces contrasting changes in calcium and calcium-binding proteins in oculomotor and hypoglossal nuclei of Balb/c mice.. J Comp Neurol.

[59] Paizs M, Engelhardt JI, Katarova Z, Siklos L (2010). Hypoglossal motor neurons display a reduced calcium increase after axotomy in mice with upregulated parvalbumin.. J Comp Neurol.

[60] Adret P, Margoliash D (2002). Metabolic and neural activity in the song system nucleus robustus archistriatalis: effect of age and gender.. J Comp Neurol.

[61] Hensch TK (2005). Critical period mechanisms in developing visual cortex.. Curr Top Dev Biol.

[62] Jarvis ED, Nottebohm F (1997). Motor-driven gene expression.. Proc Natl Acad Sci U S A.

[63] Smyth GK (2004). Linear models and empirical bayes methods for assessing differential expression in microarray experiments.. Stat Appl Genet Mol Biol.

[64] Hackett SJ, Kimball RT, Reddy S, Bowie RC, Braun EL (2008). A phylogenomic study of birds reveals their evolutionary history.. Science.

[65] Simonyan K, Horwitz B (2011). Laryngeal motor cortex and control of speech in humans.. Neuroscientist.

